# Avian Embryonic Culture: A Perspective of *In Ovo* to *Ex Ovo* and *In Vitro* Studies

**DOI:** 10.3389/fphys.2022.903491

**Published:** 2022-05-16

**Authors:** Woranop Sukparangsi, Ampika Thongphakdee, Sittipon Intarapat

**Affiliations:** ^1^ Department of Biology, Faculty of Science, Burapha University, Chon Buri, Thailand; ^2^ Wildlife Reproductive Innovation Center, Research Department, Bureau of Conservation and Research, Zoological Park Organization of Thailand Under the Royal Patronage of H.M. the King, Bangkok, Thailand; ^3^ Department of Anatomy, Faculty of Science, Mahidol University, Bangkok, Thailand

**Keywords:** avian embryo, embryonic development, *ex ovo* cultivation, *in ovo* cultivation, *in vitro* culture, pluripotency

## Abstract

The avian embryos growing outside the natural eggshell (*ex ovo*) were observed since the early 19th century, and since then chick embryonic structures have revealed reaching an in-depth view of external and internal anatomy, enabling us to understand conserved vertebrate development. However, the internal environment within an eggshell (*in ovo*) would still be the ideal place to perform various experiments to understand the nature of avian development and to apply other biotechnology techniques. With the advent of genetic manipulation and cell culture techniques, avian embryonic parts were dissected for explant culture to eventually generate expandable cell lines (*in vitro* cell culture). The expansion of embryonic cells allowed us to unravel the transcriptional network for understanding pluripotency and differentiation mechanism in the embryos and in combination with stem cell technology facilitated the applications of avian culture to the next levels in transgenesis and wildlife conservation. In this review, we provide a panoramic view of the relationship among different cultivation platforms from *in ovo* studies to *ex ovo* as well as *in vitro* culture of cell lines with recent advances in the stem cell fields.

## Introduction

In mammals, it is infeasible to observe the development of embryos outside the womb from fertilization to birth. Possibly, aves as a mammalian counterpart provide us an excellent model to be able to observe such a thing. Their advantages are low-cost without the need of feeding the embryos, ease of handling the eggs, and easy visualization of the embryonic development (Wolpert, 2004; Stern, 2005). Several studies have observed morphological changes in the development of the embryo within the eggshell by windowing or injecting compounds into the egg to observe changes in hatchability or physiological response in *in ovo* studies, or by removing the embryos from natural eggshells to new surrogate eggshells in semi-shell-less studies; however, there is a limitation in each method (Silver, 1960; Fisher and Schoenwolf, 1983; Andacht et al., 2004). Alternatively, completely removing embryos from their natural eggshells to various types of recently developed artificial vessels for further observation of developmental changes and genetic manipulation is called *ex ovo* studies. To provide in-depth studies into avian embryogenesis, parts of embryos were taken to derive cell lines in *in vitro* culture including embryonic stem cells (ESCs) and primordial germ cells (PGCs), and become an essential tool to understand pluripotency network regulating early development (van de Lavoir et al., 2006; Choi et al., 2010; Aubel and Pain, 2013; Whyte et al., 2015; Altgilbers et al., 2021). *In vitro* culture with genetic manipulation also enables us to study the gain/loss of functions and perform cellular reprogramming to produce induced pluripotent stem cells (iPSCs) capable of differentiation into all cell types, similar to ESCs (Dai et al., 2014; Katayama et al., 2018). Thus, in this review, we highlight the recent development of techniques used to study avian embryos in both *in ovo* and *ex ovo* cultivation as well as environmental parameters affecting hatchability and also the roles of *in vitro* culture to understand avian early development; in particular, pluripotency and germline-competent stem cells are also discussed.

## *In Ovo* Cultivation: Essential Tool for Avian Development Insight

The avian embryo developed within a calcium carbonate-containing eggshell to provide protection and to be part of normal development. This enclosing environment of the eggshell provides sufficient nutrients from the yolk and osmotic pressure from albumen to generate a new live young chick, unlike receiving continuing support within the mother’s body in eutherian mammals. The isolated and complete system of avian eggs to progress into full development without further needs of materials from the mother, and high availability with low cost in poultry markets enables us to easily study the development of avian species, in particular the domestic chick (*Gallus gallus domesticus*). In recent years, studies have shown the great advantages of using developing chicken eggs in biotechnology including *in ovo* delivery of biological supplements and vaccination *via* amniotic inoculation or embryo body inoculation (reviewed in Saeed et al., 2019). *In ovo* feeding also plays an important role to improve the health of gastrointestinal tracts and immunity, increasing hatchability and resistance to pathogens (reviewed in Das et al., 2021). From day 4 to 5 post laid egg, the chorioallantoic membrane (CAM), a vascularized membrane responsible for gas exchanges that attaches directly to the eggshell, develops and this *in ovo* CAM structure provides a great platform to study diverse fields (reviewed in Nowak-Sliwinska et al., 2014) including tissue engineering [e.g., biomaterial and biosensors (Borges et al., 2003; Valdes et al., 2003; Azzarello et al., 2007)], testing of various compounds in angiogenesis (Nowak-Sliwinska et al., 2014), drug screening, and tumor growth treatment (Dupertuis et al., 2015).

**FIGURE 1:**
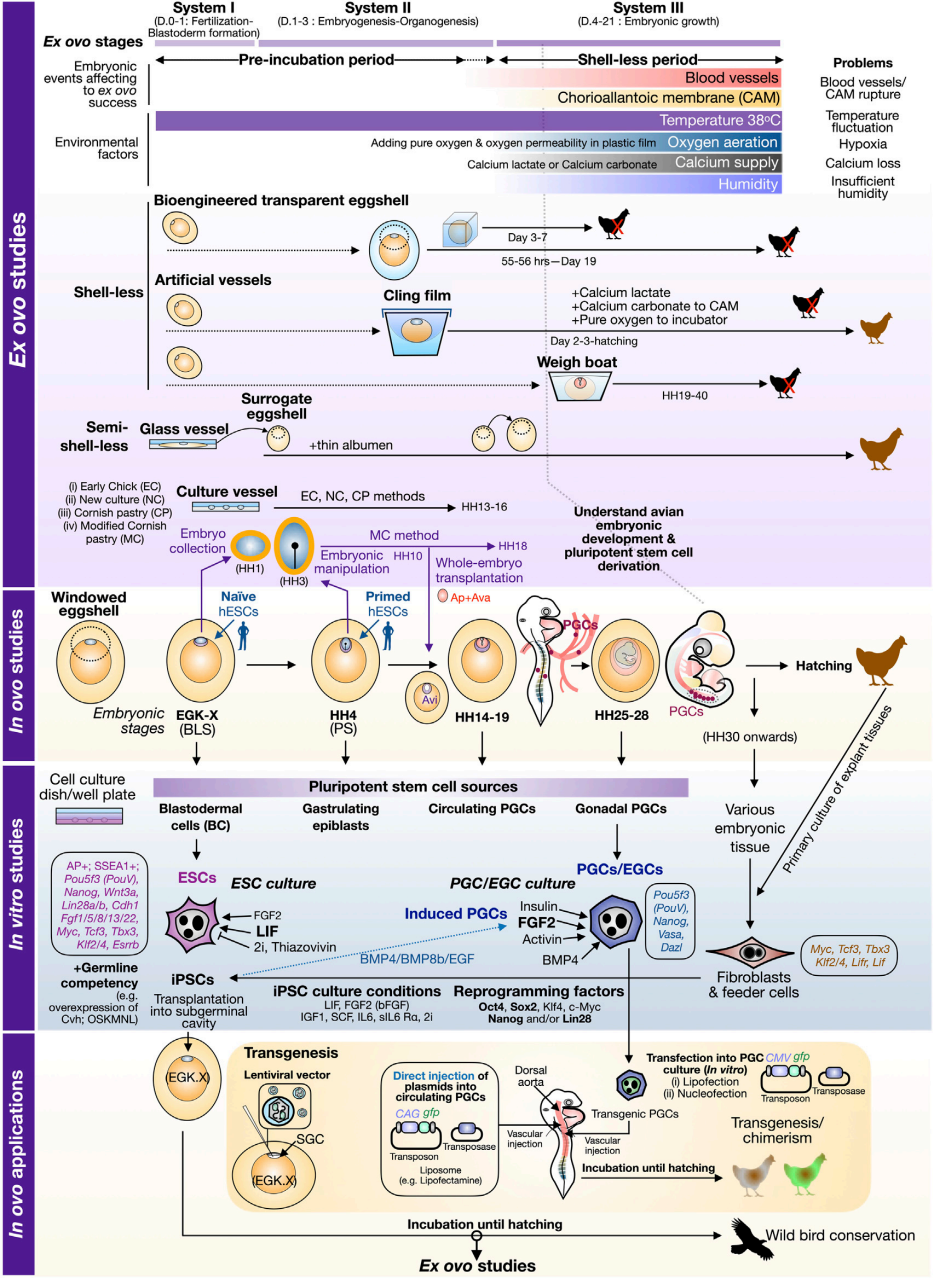
Perspective view of avian embryonic culture with *ex ovo–in ovo* cultivation and *in vitro* cell culture. In the schematic illustration of *ex ovo* studies, avian embryos at early stages can be collected into culture vessels (e.g., Petri dish) and cultured under chemically defined solutions (as listed in Table 1). In “shell-less” culture, experiments aimed to nourish embryos without natural eggshells until hatching. Specific types of shell-less cultures are described in Table 1. In “semi-shell-less” culture, early embryos are temporarily cultured in the culture vessels and later transferred to surrogate eggshells with the addition of thin albumen. A bigger eggshell is also required at the transition from embryonic system II to III. Environmental factors and potential problems affecting the success of *ex ovo* culture are noted in relation to embryonic systems I–III. *In ovo* culture depicts the early stages (EGK X to HH28, day 0–5) of avian embryos using a chick as a model. The early embryonic stage of a chick embryo at the freshly laid egg and stage HH4 can be incorporated well with naive and primed ESCs, respectively (Akhlaghpour et al., 2021). Dark magenta dots in the embryo stages HH14–19 and HH25–28 represent primordial germ cells (PGCs) circulating in blood vessels and migrating to the genital ridges which later become gonads (Nakamura et al., 2013). The embryos described in *in ovo* are also used to establish pluripotent stem cells in *in vitro* culture. Specific culture conditions to support avian ESCs/EGCs are also noted with a brief list of gene expression profiles from Katayama et al. (2018). Fibroblasts are a common source for cellular reprogramming. The list of reprogramming factors, Oct4 (O), Sox2 (S), Klf4 (K), c-Myc (M), Nanog (N), and Lin28 (L), are listed as well as cytokines and inhibitors used for avian iPSC induction. The bottom panel shows the connection from *in vitro* studies to *in ovo* works, in particular how to produce transgenic birds *via* injection of lentiviral vectors in the subgerminal cavity (SGC), transgenic PGCs, and direct transfection of plasmids into the dorsal aorta of early embryos. Brown chicks indicate embryonic progression into hatching. Black chicks with red cross indicate embryos incapable of hatching or terminated before hatching. Brown-white/brown-green chicks indicate the current success of chimera and transgenic birds. Abbreviation: AP+, positive alkaline phosphatase staining; Ap, area pellucida; Ava, area vasculosa; Avi, area opaca vitellina; D., embryonic day; EGC, embryonic germ cells; EGK, Eyal-Giladi and Kochav (1976) Chick Embryonic Stages; HH, Hamburger and Hamilton (1951) Chick Embryonic Stages; hESCs, human embryonic stem cells; GC, germline competency.

To address the mechanism of development in avian embryos, chick embryos in *in ovo* cultivation have been used to understand the changes in embryonic morphology over time through the open windowed eggshell (windowing) method (Speksnijder and Ivarie, 2000; Andacht et al., 2004; Korn and Cramer, 2007), Figure 1. The key question in development is to understand pluripotency networks orchestrating early development and how these networks are conserved across species, in particular mammalian versus avian models. The timeframe to study early events in pluripotency acquisition [indicated by the presence of *Pou5f3* (*PouV*), *Sox2*, and *Nanog*] in avian species is limited due to the onset of development that has already occurred since intrauterine development (Han et al., 2018) requires the sacrifice of hens. At the freshly laid egg stage, pluripotency has been shown to differ between finch and chick embryos, as the finch blastoderm in the laid egg (EGK V–VIII) expresses more naive-like state genes similar to naïve mouse embryonic stem cells (mESCs) while the chick blastoderm at the laid egg (EGK IX–XI) has primed bias similar to the mouse epiblast stem cells (EpiSC). This suggests that the pluripotency mechanism in different avian species is diverse at the early stage of freshly laid eggs and cannot be relied only on a chick model. A recent study of *in ovo* interspecies chimerism also shows the striking result to highlight the pluripotent state at equivalent points between human versus chick at an early embryonic development in that the EGK X blastodermal cells matched well with naïve human pluripotent stem cells (hPSCs), while the primed hPSCs can incorporate into the gastrulating epiblasts of chick embryos (Akhlaghpour et al., 2021) (Figure 1). In addition, CAM is used to understand pluripotency properties in an interspecies manner. Traditionally, the mouse model has been used as a platform for tumor formation from ESCs or induced pluripotent stem cells (iPSCs) established from various species of mammals to ensure the true nature of cells possessing “pluripotency”; the method is called teratoma assay. To replace mouse as an animal model for teratoma assay, CAM in the *in ovo* cultivation was used as a platform for seeding human iPSCs, which can grow on CAM and form a three-germ layer containing tumors within 9 days at 37°C (Weber et al., 2021). Taken together, *in ovo* study is still an essential tool for an ideal environment to unravel the developmental state and can be used to understand mammalian development mechanisms to reduce the use of animals, in particular mice, in the 3R model.

## *Ex Ovo* Cultivation of Avian Embryos: Environmental Parameters for Successful Hatching

*Ex ovo* culture is a system in which the original eggshell is removed, and the embryos are transferred to the new culture milieus, including a surrogate eggshell, Petri dishes, and artificial eggshell-like vessels (Ono, 2000; Borwompinyo et al., 2005; Liu et al., 2012; Tahara and Obara, 2014). By removing the eggshells, the *ex ovo* culture allows us to manipulate developing embryos at certain stages for surgical methods (Cloney and Franz-Odendaal, 2015). Of course, it also paves the way to seek how the embryo develops without a protective layer (Buskohl et al., 2012). Many studies have used this technique in several aspects such as angiogenesis (Hockel et al., 1987), intravasation assays (Uchibayashi et al., 1992), grafting, and tumor formation (Dohle et al., 2009; Villanueva and Sikora, 2022). Moreover, this technique can be applied to the lessons in high school and advanced developmental biology research fields (Buskohl et al., 2012; Dorrell et al., 2012; Cloney and Franz-Odendaal, 2015).

Since a previous study has shown that metabolism and internal environment within eggshells change dramatically at different stages (Givisiez et al., 2020), *ex ovo* cultivation aimed to follow embryonic development until hatching needs to find the optimal balance of several factors, in particular oxygen demand, humidity, and calcium requirement. Generally, the development of chick embryos can be divided into three phases: fertilization to blastoderm formation (day 0–day 1), embryogenesis (day 1–day 3), and embryonic growth (day 4–day 21) (Perry, 1988). Hitherto, three different aforementioned systems can be categorized into three systems with roman numerals: system I (for day 0–day 1), system II (day 1–day 3), and system III (day 4–day 21). The details of different techniques or modified methods described in previous studies are summarized in Table 1. Here, we also provide insights into each parameter (i–v) required for successful *ex ovo* studies and hatchability in relation to three phases of avian development (Figure 1).

**TABLE 1:** Timeline of *ex ovo* techniques developed to understand the embryonic development in avian species.

*Ex ovo* Technique	Species	Objectives and key benefits	References
**Embryonic manipulation at early stages**			
Watch-glass	Chick and duck	To cultivate an avian blastoderm on a clot of fowl plasma and embryo extract which remained alive for 2–3 days	Waddington (1932)
Albumen-agar	Chick	To explant the blastoderm on a semi-solid substratum containing clot with more stiffness by agar and diluted albumen	Spratt (1947)
New culture	Chick	To support the explant and expansion of the blastoderm up to 48 h of incubation (appearance of primitive streak and blood circulation)	New, (1955)
Early chick (EC) culture	Chick	To culture the whole embryo using a filter paper carrier	Chapman et al. (2001)
Cornish pasty	Chick and quail	To grow chick and quail embryos from stage 3HH to stage 18HH with normal morphology	Connolly et al. (1995); Nagai et al. (2011)
**Semi-shell-less**			
Surrogate eggshell	Domestic fowl and turkey	To be able to transfer the cultured embryos into different species	Rowlett and Simkiss (1987)
**Shell-less**			
Baggie	Chick	To grow the embryos in artificial membranes using polyethylene bags	Elliott and Bennett (1971)
Petri dishes	Chick	To grow the embryos in Petri dishes from the 3rd to the 20th day of incubation	Auerbach et al. (1974)
The plastic wrap/culture tripod	Chick	To be able to access the embryo and its membranes for experiments	Dunn and Boone, (1976)
Beaker	Chick	To observe the embryos from the unincubated stage to the 19th day of incubation	Barnett (1982)
Modified Callebaut’s method	Chick and quail	To investigate development events of the single-cell stage fertilized egg taken from the maternal oviduct	Perry (1988)
Polytetrafluoroethylene (PTFE) membrane	Quail	To use a gas-permeable membrane to increase the viability of culture embryos	Kamihira et al. (1998)
Polyurethane (PU) membrane	Chick	To culture whole embryos on hexagonal weigh boats for up to 10 days	Yalcin et al. (2010)
Polymethylpentene (PMP) film	Chick	To establish a simple method for culturing the embryos with high hatchability using a plastic film	Tahara and Obara (2014)
**Egg-in-cube** (polydimethylsiloxane: PDMS and polycarbonate: PC)	Chick	To generate artificial transparent eggshell with functionalized surface allowing better observation of chick embryo development	Huang et al. (2015)
**Bioengineered transparent eggshell** (polydimethylsiloxane: PDMS polymer)	Chick	To generate artificial transparent eggshell retaining a natural shape that can support normal chick development; enhance the visibility of imaging in 3D	Ishak et al. (2020)

### Incubation Period and Embryonic Stage

Significantly, the embryonic age is the key factor for manipulating *ex ovo* cultured embryos. To increase viability during culture, transferring the embryos to the new culture vessels after stage HH15-16 (Hamburger and Hamilton, 1951) is recommended (Tahara and Obara, 2014). Those embryos developing in a polymethylpentene film and supplemented with calcium lactate and distilled water resulted in a 90% survival rate (Tahara and Obara, 2014). Tahara and Obara (2014) also showed that the embryos transferred to the culture vessel after stage HH16 showed viability on day 8 of incubation. Hence, the timing of the incubation period before transferring to the new environment plays a major role in enhancing the viability. Ideally, the embryos should be transferred to the new culture vessel at stage HH19 (Rowlett and Simkiss, 1989; Borwompinyo et al., 2005). Even though different egg preincubation periods between stages HH13 and 16 affected embryonic survival, higher viability was still observed before stage HH15 (Tahara and Obara, 2014). In addition to the embryonic age, it was also reported that care had to be taken with the vitelline membrane as it could be easily damaged while transferring to the new culture vessel (Tahara and Obara, 2014). This can be seen particularly in manipulating after-stage HH17 embryos. Dohle et al. (2009) reported that the stage HH35 (at around 9 days of incubation) embryos were less sensitive to agitation when compared to those at earlier stages. In addition, the survival rate of *ex ovo* cultured embryos can be higher up to 90–100% when they reached stage HH35, but then declined at the later stages, that is at stages HH40–41 (Cloney and Franz-Odendaal, 2015). Altogether, handling the right time of incubation period to acquire the right embryonic stage to proceed with hatching is essential for the *ex ovo* culture technique.

### Temperature and Humidity

Practically, the temperature inside the incubator should not be lower than 38°C, which could cause a developmental delay (Dohle et al., 2009), and the ideal humidity was reported at 40% (Cloney and Franz-Odendaal, 2015). However, it was reported that covering the top of culture vessels with a polystyrene plastic lid could maintain 100% humidity and could be varied to 38°C with 80% humidity (Tahara and Obara, 2014). When the CAM is formed, which can be recognized on day 3 of incubation, the humidity is vital to preventing the desiccation of embryonic structures (Dohle et al., 2009). Importantly, the humidity of the system at the stage of the yolk sac formation is crucial to decreasing the humidity, to lower than 60%, which caused the CAM rupture by sticking to the eggshells (Dohle et al., 2009). Regarding the concern with water circulation systems, Yalcin et al. (2010) recommended that the culture should be kept at an optimal temperature. The design of the vessel structure is not only to prevent water loss but to facilitate oxygen supply (Tahara and Obara, 2014). The installation of a circulating water bath within the culture system helps to maintain the incubation temperature for developing embryos (Yalcin et al., 2010). In addition, a resistance heater was also used to regulate the water temperature (Yalcin et al., 2010).

### Calcium Supplementation

Calcium, primarily calcium carbonate, is one of the major constituents of the eggshells (Scanes and Christensen, 2020) that serves as a tertiary envelope to protect developing embryos (Bellairs and Osmond, 2014). The majority of calcium provided to the embryos until hatching derives from the eggshell (Rowlett and Simkiss, 1987; Kamihira et al., 1998). Furthermore, calcium from the eggshells is also needed for ossification, and its deficit results in limb deformity (Cloney and Franz-Odendaal, 2015); therefore, supplying calcium to developing embryos is required (Elliott and Bennett, 1971; Rowlett and Simkiss, 1987). During the *ex ovo* culture, the embryos develop with no eggshells as the protective structure leading to a high mortality rate (Tahara and Obara, 2021). Therefore, adding calcium carbonate to the CAM in shell-less cultured embryos helped to increase more than 40% hatchability (Tahara et al., 2021). Kamihira et al. (1998) reported that various forms of calcium including eggshell powder and calcium lactate could also be added to the albumen as a calcium source following the eggshell removal. Although calcium carbonate is the main substance found in the eggshells, it failed to increase the hatchability rate for the shell-less system (Tahara et al., 2021). Alternatively, calcium lactate was shown to improve the hatchability rate with less toxicity to the cultured embryos (Kamihira et al., 1998; Tahara and Obara, 2014). However, supplementing calcium carbonate could increase hatchability up to 40% if it was added to the CAM (Tahara et al., 2021). These indicate that calcium supplementation with the minimum requirement is necessary for developing embryos to hatch in the shell-less system. Furthermore, information to understand the details of calcium availability and dynamics during embryonic development in this system still needs functional and molecular studies.

### Oxygen Supply

Aeration in the culture vessels is mandatory for the survival of the embryos due to the loss of moisture by embryonic transpiration (Dohle et al., 2009). Importantly, the efficiency of the hatching rate can be improved by adding pure oxygen to culture vessels (Kamihira et al., 1998; Tahara and Obara, 2014). Previous studies reported that the number of embryos survived at the later stages in the pure oxygen culture system is higher than 50%, indicating the achievement of a high hatchability by culturing in such an artificial vessel (Kamihira et al., 1998; Tahara and Obara, 2014). Practically, the aeration in this culture system can be made to the ventilation holes on the top of the sealing film (Tahara and Obara, 2014). Ideally, the rate of aeration with pure oxygen installed plastic tube in the vessel should be 500 ml/h, and it should be well prepared, as the embryos do not survive to hatch without an oxygen supply (Tahara and Obara, 2014). Rowlett and Simkiss (1989) reported that chick embryos cultured in an artificial vessel made of a “cling-film” were susceptible to hypoxia and hypocapnia. Moreover, oxygen aeration at the early stages of culture should be a concern due to its toxic effect that can reduce embryonic viability (Tahara and Obara, 2014). Considerably, for the later stages (stage HH43–44), at around 17 days of incubation, oxygen insufficiency can be noticed by changing the color of the CAM vessels (Dorrell et al., 2012; Tahara and Obara, 2014; Tahara et al., 2021), indicating that oxygen aeration to the later stages is indispensable for the *ex ovo* culture.

### Sealing Film

Favorably, in the *ex ovo* culture, the use of a transparent plastic film allows the embryos to reside as a cradle, namely, a hammock (Kamihira et al., 1998; Yalcin et al., 2010; Tahara and Obara, 2014) as mentioned in Table 1. The adhesive property of this material with the culture vessels is relatively low. A rubber band was carefully used to tighten the film with culture vessels firmly averting embryonic mortality during manipulation (Cloney and Franz-Odendaal, 2015). A previous study reported that the positioning of the eggs and the sealing material in culture vessels affect the hatchability rate (Borwompinyo et al., 2005); the sealing materials including Handi and Saran plastic films were able to increase the percentage of hatchability (Borwompinyo et al., 2005). There are different types of plastic packaging such as polyethylene, polyvinylidene chloride, and polymethylpentene. Tahara and Obara (2014) demonstrated that these materials should possess oxygen permeability. One important point that also increases viability and hatchability is smoothening of film surface since film wrinkles caused a low survival rate of culture embryos (Tahara and Obara, 2014, 2021). Although using transparent films would help to facilitate whole-embryo observation, some disadvantages of their properties should be ameliorated to increase the hatchability rate.

## *In Vitro* Culture: Essential Toolkit for Avian Pluripotency Studies, Transgenesis, and Conservation

As pluripotent cells in embryos are restricted in some periods and in some embryonic tissues, the understanding of the pluripotency mechanism in embryos is limited. The advents of *in vitro* culture from avian embryos enable us to examine the pluripotency network and possible use of germline-competent stem cells (GCSCs) in avian biotechnology, in particular gene editing, transgenesis, and wild bird conservation (Han et al., 2015). There are four main types of GCSCs that can be derived from the *in vitro* culture of avian embryonic tissues: blastodermal cells/embryonic stem cells (ESCs), embryonic germ cells (EGCs), primordial germ cells (PGCs), and spermatogonial stem cells (SSCs) (Han et al., 2015). The discovery of chick ESCs and blastodermal cell culture emphasizes the conserved network regulating pluripotency required at least leukemia inhibitory factor (LIF) (Etches et al., 1997; Pain et al., 1996), a cytokine used to culture naïve mouse ESCs and iPSCs (Niwa et al., 1998; Takahashi and Yamanaka, 2006). In addition, basic fibroblast growth factor (bFGF), a cytokine used to cultivate primed human ESCs/iPSCs (Thomson et al., 1998; Takahashi et al., 2007), is required in several avian ESCs/iPSCs studies (Pain et al., 1996; van de Lavoir et al., 2006; Whyte et al., 2015; Choi et al., 2016; Katayama et al., 2018). Thus, this suggests that pluripotent stem cells from avian species exhibit some bias in a naïve-primed direction which could depend on other culture supplements. Several studies have used (Aubel and Pain, 2013) protocol to culture chick iPSCs in DMEM/F12 containing other several cytokines in addition to LIF and/or bFGF (FGF2) including IGF1, SCF, IL6, and sIL6 Rα (Dai et al., 2014; Fuet and Pain, 2017; Fuet et al., 2018). To force pluripotent cells to bias toward a naïve state, two inhibitors’ (2i) cocktail including CHIR99021 (GSK3 inhibitor) and PD0325901 (MEK inhibitor), generally used in naïve mouse ESCs (Ying et al., 2008) can also support the establishment of chick iPSCs (Dai et al., 2014; Katayama et al., 2018; Yuan et al., 2021), as shown in Figure 1. In avian cellular reprogramming, choices of delivery and expression system and reprogramming factors are crucial for induction success of iPSCs. Exogenous gene delivery can be done *via* using a virus (retrovirus, lentivirus, and Sendai virus) (Dai et al., 2014; Fuet et al., 2018) and liposome-based transfection of a piggyBac transposon carrying a single polycistronic reprogramming cassette (Modified Oct4, Sox2, Klf4, c-Myc, Lin28, and Nanog) (Katayama et al., 2018) (Figure 1). Reprogramming factors used for induction of avian iPSCs varied in several studies but at least Oct4 and Sox2 were used in all studies (Dai et al., 2014; Fuet and Pain, 2017; Fuet et al., 2018; Katayama et al., 2018), while Klf4 and c-Myc were replaced with Nanog and Lin28 in Yuan et al.( 2021) and Zhao et al. (2021). Fuet et al. (2018) also showed that Nanog is essential for long-term iPSC culture. In addition to pluripotent stem cells, *in vitro* culture allows the exploration of the primordial germ cell (PGCs) specification mechanism. It has been shown that avian PGCs do not require LIF for self-renewal (Whyte et al., 2015) while mammalian PGCs need it (Leitch et al., 2013). But, instead, bird PGCs, which resemble more the mammalian gastrulating epiblast (or *in vitro* mouse EpiSCs), can be supported under FGF2, insulin, and activin-BMP4 to induce SMAD signaling (Whyte et al., 2015). Also, chick iPSCs can differentiate into induced PGCs (iPGCs) which can be transplanted into another strain of a chick embryo to produce viable offspring (Zhao et al., 2021).

*In vitro* culture also supports the study of transcriptomic analysis of established ESCs/iPSC cell lines and sheds light on the conservation and uniqueness of pluripotency-related transcriptional networks in the avian species (Jean et al., 2015; Katayama et al., 2018). The list of ESCs/PGCs versus fibroblast markers is also shown in Figure 1. Finally, the key advantage of *in vitro* culture is the application of established cell lines including GCSCs back into *in ovo* cultivation for producing interspecific avian chimeras (within avian species or even mammalian to avian species), as shown in Figure 1. At the early stage of transgenic avian technology, transgenesis in chick embryo was done using a virus-dependent approach, e.g., injection of lentiviral vectors into the subgerminal cavity of the early embryo (Chapman et al., 2005). The advent of PGC culture allows easy manipulation of gene delivery *via* transfection (lipofection or nucleofection) as shown in the success of stable integration of *gfp* transgene into the host genome and later transgenic PGCs can be transplanted back to the embryos to eventually generate chimera and transgenic chicks (Park and Han, 2012). Recently, the direct injection of plasmids carrying the gene of interest, together with a liposome-based reagent, into the dorsal aorta of chick embryo containing circulating PGCs was performed with the successful production of transgenic quail offspring (Serralbo et al., 2020). Overall, the achievement of *in vitro* culture with *in ovo* application and avian embryo plasticity to accept xenograft cells provide a hope of using chick embryos as a platform to propagate endangered wild birds under stem cell-based conservation.

## Future Recommendations and Conclusion

For *ex ovo* milieu, exploration of possible avenues on how to culture avian embryo without a natural shell continues with the further development of imaging, chemistry, and nano-biomaterial technology, which can provide better-engineered eggshells resembling natural ones and fit to in-demand experiments such as in-depth imaging of embryos or transgenic embryos with artificial eggshells intact. In addition to *in vitro* culture, research on cellular reprogramming and ESCs derivation of various avian species is still required to understand the nature of naïve-primed pluripotency in relation to germline competency-the benefit of this understanding is that one day it can provide long-term security and prevent the extinction of avian species.
